# Applicability and performance evaluation of the Shennong 1 chicken 40 K liquid chip in commercial chicken populations

**DOI:** 10.1016/j.psj.2026.106918

**Published:** 2026-04-09

**Authors:** Zhao Cai, Shu Wang, Chaoqun Gao, Chenpeng Xiao, Yulong Ma, Yunjuan Liu, Kejun Wang, Yadong Tian, Xuewei Fan, Hangran Gao, Guirong Sun, Xiangtao Kang, Wenting Li

**Affiliations:** a The Shennong Laboratory, College of Animal Science and Technology, Henan Agricultural University, Zhengzhou, 450046, China; b Henan Fengyuan Poultry Industry Company Limited, Nanyang 473003, China

**Keywords:** Shennong 1 chicken 40 K liquid chip, Whole-genome resequencing, Commercial chickens, Performance evaluation

## Abstract

To evaluate the applicability and analytical performance of the Shennong 1 Chicken 40 K Liquid chip (Shennong chip) in commercial chicken populations, parental lines of Australorp (Arp) and Rhode Island Red (RIR) chickens were analyzed using both whole-genome resequencing (WGS) and Shennong chip genotyping. A total of 128 individuals were included. The performance of Shennong chip was evaluated by comparing its results with those of WGS through population genetics analyses, including assessment of population structure, genetic diversity and selection signatures. Across analyses, Shennong chip showed high concordance with WGS in population genetic inference. For example, the first two principal components derived from the two datasets showed nearly identical distributions, with Pearson correlation coefficients close to 0.99. Similar cluster pattern was observed between WGS and Shenong chip results in ancestry inference at K = 2 and K = 3. Genetic diversity estimates suggested moderate levels of variation (Shennong chip: He ≈ 0.34–0.40; WGS: pi ≈ 0.0027–0.0037) across all four parental lines, with relatively higher diversity(He ≈ 0.40; pi ≈ 0.0037) observed in the Rhode Island Red D line. The SNP chip reliably captured major population structure patterns, genetic diversity differences among parental lines, and biologically interpretable selection signals. Functional enrichment analyses based on SNP chip–derived candidate regions further revealed distinct selection orientations between parental lines. LSBL analysis identified Arp-specific selection signals, while joint *F*__ST_ and nucleotide diversity analyses revealed pronounced divergence between RIR C and D. Functional enrichment highlighted neural signaling–related genes in RIR C (e.g., *GRIK1*) and metabolic and immune-related genes in RIR D (e.g., *AvBD* genes) Overall, these results demonstrate that SNP chip provides a reliable, cost-effective, and scalable alternative to WGS for population genetic and breeding-oriented genomic analyses in commercial chicken populations.

## Introduction

With the rapid development of genomic technologies, studies on the genetic basis of economically important traits in livestock and poultry have gradually shifted from traditional molecular markers to genome-wide approaches (Zhou et al., 2022). In chickens, genome-wide single nucleotide polymorphism (SNP) markers have been increasingly applied in population genetic analyses, genetic evaluation, and breeding programs, contributing to improved breeding efficiency (Song et al., 2023) and supporting sustainable poultry production (Yang et al., 2021). Consequently, obtaining genome-wide SNP information in an efficient, accurate, and cost-effective manner has become a key requirement in modern poultry breeding systems (Marchesi et al., 2021).

At present, genome-wide SNP datasets are primarily generated using whole-genome resequencing (WGS) or SNP chip–based genotyping platforms. WGS provides comprehensive coverage of genomic variation and enables the detection of rare and population-specific variants (Zhang et al., 2025). However, its relatively high cost, large data volume, and computational demands still limit its routine application in large-scale breeding populations. In contrast, SNP chips offer advantages in genotyping efficiency, cost control, and analytical stability, and have therefore been widely adopted in animal breeding, population history inference, and genetic diversity studies (Auvray et al., 2014; Muñoz et al., 2019).

According to the National Catalogue of Livestock and Poultry Genetic Resources of China (2024 edition), 140 indigenous chicken breeds have been officially recognized, characterized by substantial diversity in morphology, production traits, and environmental adaptability (Luo et al., 2020; Tian et al., 2025). However, the development of SNP chips tailored for indigenous chickens faces several challenges. Individual indigenous breeds often have relatively small population sizes, while genetic diversity among breeds is high. These features complicate SNP selection and require careful balancing between population specificity and cross-breed applicability. In recent decades, the rapid expansion of high-yield commercial breeds has further intensified pressure on indigenous chicken resources, leading to genetic erosion and reduced utilization of local breeds (Cendron et al., 2020; Zhang et al., 2019). Under these circumstances, the development of SNP chips with both high resolution for indigenous populations and broad applicability across diverse genetic backgrounds is essential for genetic conservation, resource evaluation, and sustainable utilization (Li et al., 2024).

To address this need, we previously developed the Shennong 1 Chicken 40 K Liquid chip (Shennong chip), which was designed based on genomic characteristics of Chinese indigenous chicken populations. Validation using 204 individuals from seven local breeds showed a high average target capture rate (99.474%), and most loci (98.557%) exhibited a minor allele frequency (MAF) greater than 0.05. Analyses based on this chip were able to clearly separate different breeds, and the inferred population structure was consistent with known breed classifications, indicating good resolution and applicability. In addition, selection signature analyses identified several candidate genes related to production performance and environmental adaptation, including IGF2BP1 (Wang et al., 2021, 2024), SOX5, CACNA1G, and CXCR4. The inclusion of functional loci in the chip design further supports its use in the evaluation of economically important traits (Gao et al., 2025).

In another scenario, indigenous chicken resources are frequently utilized through crossbreeding with commercial lines in practical breeding systems. Commercial breeds play a central role in modern poultry genetic improvement. Whether our Shennong chip can be effectively applied to commercial chicken populations therefore remains an important question. In this study, the Shennong chip was applied to two representative commercial chicken breeds, Australorp (Arp) and Rhode Island Red (RIR). By integrating Shennong chip and WGS data, we conducted comparative analyses of population structure, genetic diversity and selection signatures. The objective of this study was to evaluate the analytical performance and applicability of Shennong chip in commercial chicken populations and to provide empirical evidence for its broader use in poultry breeding and genetic monitoring programs.

## Materials and methods

### Sample collection

A total of 128 individuals were collected from a commercial breeding company in Henan Province, China, representing four parental breeding lines from two commercial chicken breeds: Arp and RIR (Table 1).

**Table 1:** Sample composition of Arp and RIR parental lines.

Population	Line	Sex	Number
Arp	A	Male	20
Arp	B	Male	20
Arp	B	Female	14
RIR	C	Male	20
RIR	C	Female	14
RIR	D	Male	20
RIR	D	Female	20

Specifically, the Arp population included line A, consisting of 20 males, and line B, consisting of 20 males and 14 females. The RIR population included line C, consisting of 20 males and 14 females, and line D, consisting of 20 males and 20 females. All sampled individuals were unrelated at the pedigree level according to breeding records and were maintained under standardized management conditions.

Each individual was subjected to both whole-genome resequencing (WGS) and genotyping using the Shennong chip. For WGS, genomic DNA was sequenced on the BGISEQ-500 platform (BGI, Shenzhen, China), with an average sequencing depth of approximately 10 × per individual. Clean reads were aligned to the chicken reference genome (GRCg7b) using BWA, and variant calling was conducted following standard pipelines. For Shennong chip genotyping, the same DNA samples were analyzed using the Shennong 1 Chicken 40 K Liquid chip according to the standard protocol. The chip was developed based on genomic characteristics of Chinese indigenous chicken populations and contains approximately 40,000 target loci distributed across the genome, with high capture efficiency and a high proportion of polymorphic markers. Two corresponding variant datasets were generated and used for parallel downstream analyses.

Although sample sizes varied among lines, all groups included a moderate number of individuals (n = 20–40), which was sufficient to support the population genetic analyses performed in this study.

### Data quality control

Quality control of SNP markers was performed separately for the WGS and Shennong chip datasets using PLINK v1.9 (Purcell et al., 2007), with identical filtering criteria applied to both datasets to ensure comparability. SNPs located on sex chromosomes and unplaced scaffolds were excluded to avoid potential bias related to sex-specific inheritance patterns, hemizygosity, and recombination differences. Markers with a missing genotype rate greater than 1% were removed, and individuals with more than 5% missing genotypes were excluded.

To minimize the potential impact of linkage disequilibrium (LD) on population structure and diversity analyses, LD pruning was conducted using a sliding-window approach with a window size of 50 SNPs, a step size of 5 SNPs, and an LD threshold of r^2^ = 0.2.

After quality control, 14,232,675 high-quality biallelic SNPs were retained in the WGS dataset, while 38,722 high-quality biallelic SNPs were retained in Shennong chip dataset. The large difference in SNP density reflects the inherent characteristics of WGS and array-based genotyping platforms.

### Data analysis

#### Population structure analysis

Population genetic structure was investigated using principal component analysis (PCA), phylogenetic tree reconstruction, and ancestry inference. PCA was performed based on genome-wide SNP data using PLINK to summarize major axes of genetic variation among individuals.

Genetic distance matrices were calculated and used to construct phylogenetic trees in MEGA X using the neighbor-joining method. Branch support was evaluated using 1,000 bootstrap replicates, and the resulting trees were visualized and annotated using the Interactive Tree of Life (iTOL) platform.

Population ancestry was inferred using ADMIXTURE v1.3 (Patterson et al., 2012). The number of ancestral populations (K) was tested from 1 to 10. For each K value, cross-validation (CV) error was calculated, and the model with the lowest CV error was considered optimal for interpretation.

#### Genetic diversity analysis

Genetic diversity within each breeding line was assessed using multiple complementary indices. Observed heterozygosity (Ho), expected heterozygosity (He), and minor allele frequency (MAF) were calculated using PLINK based on autosomal SNPs.

Genome-wide nucleotide diversity (π) was estimated using VCFtools (v0.1.16) (Danecek et al., 2011). Sliding-window analyses were performed with a window size of 100 kb and a step size of 30 kb to characterize fine-scale variation in nucleotide diversity across the genome and to facilitate comparisons between populations (Mastrangelo et al., 2023).

#### Selection signature analysis

Selection signatures were investigated using complementary strategies in Arp and RIR populations based on genome-wide SNP data.

##### LSBL-based branch-specific selection analysis in Arp

To identify selection signals specific to Arp while minimizing background divergence, locus-specific branch length (LSBL) analysis was performed by integrating pairwise *F*__ST_ estimates among populations. LSBL values were calculated according to:LSBL_AB = (*F*__ST(Arp–C)_ + *F*__ST(Arp–D)_ − *F*__ST(C–D)_) / 2 (Dhakal et al., 2024), where X denotes the focal population and Y and Z represent reference populations. LSBL was calculated using non-overlapping windows of 100 kb (window size = 100 kb; step size = 100 kb). Genomic windows within the top 5% of the genome-wide LSBL distribution were defined as candidate regions under selection.

##### Population differentiation and nucleotide diversity analysis between RIR C and D

Genomic differentiation between RIR C and D was assessed using the fixation index (*F*__ST_), and nucleotide diversity (π) was estimated separately for each line. Both statistics were calculated using a sliding-window approach with a window size of 100 kb and a step size of 30 kb. Genomic regions showing elevated *F*__ST_ together with extreme π ratios between C and D were considered candidate regions under divergent selection.

##### Functional annotation and enrichment analysis

Genes located within candidate regions (top 5%) identified from both approaches were annotated and subjected to Gene Ontology (GO) and Kyoto Encyclopedia of Genes and Genomes (KEGG) enrichment analyses using the R package clusterProfiler (version 4.14.6) with the org.Gg.eg.db annotation database. Enriched terms with adjusted *P* < 0.05 were considered statistically significant.

## Results

### Population structure analysis

**Fig. 1:**
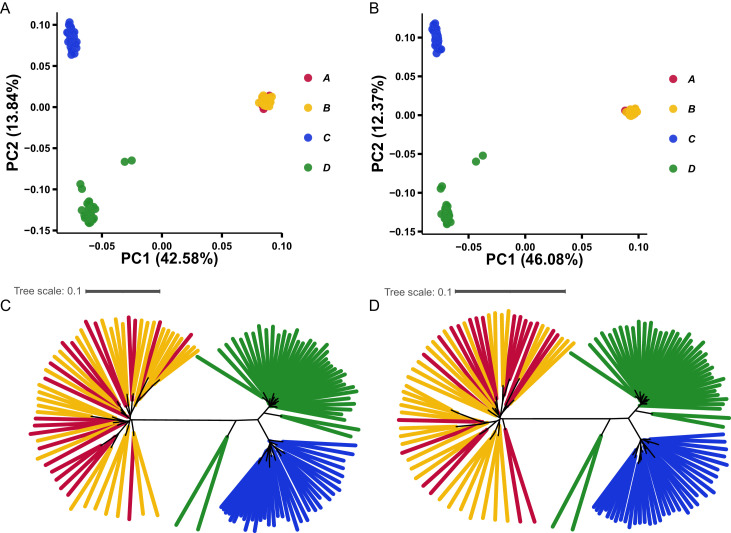
Population structure analyses based on Shennong chip and WGS. (A, B) PCA of the four parental populations based on Shennong chip (A) and WGS (B). (C, D) Phylogenetic trees constructed using individual genetic distances based on Shennong chip (C) and WGS (D).

Population genetic structure was examined using both Shennong chip and WGS datasets. Overall, the two platforms produced highly consistent patterns, allowing a robust comparison of genetic relationships among breeds and parental lines (Fig. 1A–D).

Principal component analysis (PCA) revealed clear contrasts between the Arp and RIR populations. Individuals from Arp lines A and B clustered tightly and largely overlapped on both PC1 and PC2, showing no obvious separation between the two lines (Fig. 1A–B). This overlapping distribution was consistently observed in both Shennong chip–based and WGS-based PCA results, indicating that the two Arp parental lines share a highly similar genomic background at the population level. In contrast, individuals from RIR lines C and D were clearly separated along PC2, indicating substantial genetic differentiation between these two parental lines, and this separation pattern was also concordant between the two genotyping platforms.

Phylogenetic tree reconstruction further supported the PCA results (Fig. 1C–D). Individuals from Arp lines A and B were intermingled across the tree and did not form distinct clades, consistent with their close genetic relationship inferred from PCA. By contrast, RIR lines C and D formed two relatively independent branches, supporting genome-wide divergence between the two lines. Several individuals from the RIR D line appeared as outliers on the phylogenetic trees and showed a more dispersed distribution in PCA space in both the WGS- and Shennong chip–based analyses, indicating greater within-line dispersion for RIR D than for the other lines. Taken together, PCA and phylogenetic analyses consistently indicated genetic homogeneity in Arp A and B, as well as pronounced differentiation between RIR C and D. Notably, higher within-line dispersion was observed in RIR D.

### Ancestry inference using ADMIXTURE

**Fig. 2:**
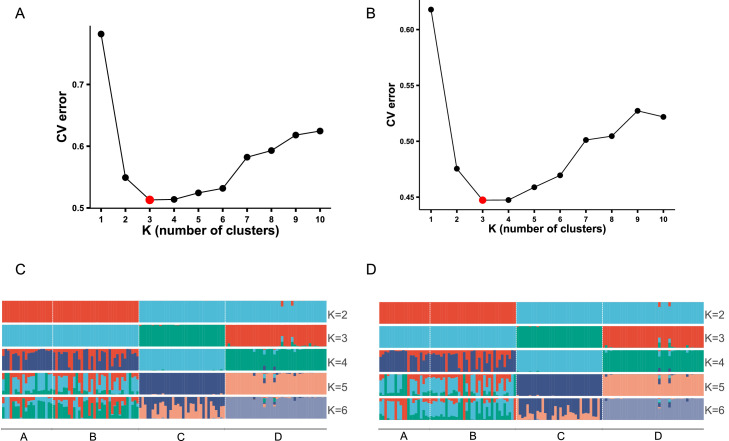
ADMIXTURE analysis based on Shennong chip and WGS. (A, B) CV error values for K = 1–10 inferred from Shennong chip (A) and WGS (B). (C, D) Individual ancestry proportions inferred at different K values based on Shennong chip (C) and WGS (D).

ADMIXTURE analysis was performed to further characterize ancestral composition across the sampled populations based on Shennong chip and WGS datasets (Fig. 2A–B). Cross-validation analysis indicated that the lowest prediction error was obtained at K = 3, suggesting that three ancestral components best explained the genetic structure.

At K = 2 (Fig. 2C–D), Arp and RIR individuals were separated into two major genetic clusters. When K increased to 3, a clear distinction emerged between RIR lines C and D, whereas Arp lines A and B remained grouped together and displayed nearly identical ancestry proportions. Importantly, the major stratification patterns observed at K = 2 and K = 3 were highly consistent between Shennong chip and WGS, supporting stable inference of the main ancestral components across platforms.

### Consistency between Shennong chip– and WGS-derived PCA

Based on the PCA results described above, a quantitative comparison was conducted to evaluate the consistency of population structure inferred from Shennong chip and WGS data. For each individual, scores of the first (PC1) and second (PC2) principal components were extracted from both datasets and subjected to pairwise correlation analysis.

**Fig. 3:**
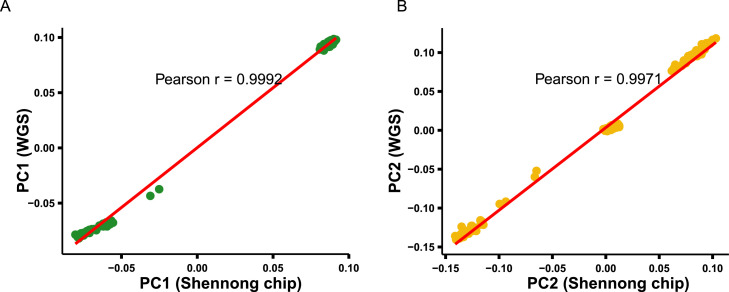
Concordance of PCA results between Shennong chip and WGS. (A) Correlation between PC1 values inferred from Shennong chip and WGS. (B) Correlation between PC2 values inferred from Shennong chip and WGS. Strong linear correlations (Pearson’s r ≈ 0.99) indicate high consistency between the two platforms.

As shown in Fig. 3A (PC1) and Fig. 3B (PC2), the two genotyping platforms exhibited a high degree of concordance in principal component space. PC1 scores displayed an almost perfect linear relationship between Shennong chip and WGS datasets, with a Pearson’s correlation coefficient (r) of approximately 0.9992, indicating that the major axis of genetic variation was consistently captured by both platforms. PC2 scores showed a similarly strong positive correlation (r ≈ 0.9971).

In addition, individuals from the same breeding lines occupied comparable relative positions and displayed consistent clustering patterns in PCA space across the two datasets. Together, these results demonstrate that Shennong chip can accurately recapitulate the population genetic structure revealed by WGS at the level of principal component analysis.

### Genetic diversity analysis

Genetic diversity in the Arp and RIR parental populations was evaluated using both Shennong chip and WGS datasets. Summary statistics derived from the two genotyping platforms are presented in Table 2.

**Table 2:** Genetic diversity indices of four parental lines estimated from Shennong chip and WGS data.

Line	Number	Shennong chip				WGS			
		He	Ho	Pi	MAF	He	Ho	Pi	MAF
A	20	0.3485	0.3143	0.0000129	0.2483	0.2969	0.2663	0.003071	0.1613
B	34	0.3869	0.2676	0.0000147	0.2936	0.2963	0.2089	0.003442	0.1829
C	34	0.3410	0.3192	0.0000117	0.2209	0.3015	0.2834	0.002711	0.1445
D	40	0.3972	0.3241	0.0000154	0.3109	0.2915	0.2415	0.003693	0.1933

Based on Shennong chip results (Table 2), observed heterozygosity (Ho) and expected heterozygosity (He) showed similar trends across the four parental lines. Among them, RIR line D exhibited the highest level of genetic diversity (He = 0.3972; MAF = 0.3109), whereas Arp lines A (He = 0.3485) and B (He = 0.3869), as well as RIR line C (He = 0.3410), showed relatively lower diversity levels.

Analyses based on WGS data (Table 2) yielded broadly consistent patterns. In particular, lines B and D showed higher nucleotide diversity (π = 0.003442 and 0.003693, respectively), while line C consistently exhibited lower values of both MAF and π across genotyping platforms.

Overall, comparison between Shennong chip and WGS datasets indicated that: (i) estimates of π and MAF derived from WGS were generally slightly higher than those obtained from Shennong chip; (ii) the relative ranking of genetic diversity among the four parental lines was highly consistent between the two platforms; and (iii) RIR line D displayed relatively higher genetic diversity, whereas line C showed comparatively lower diversity in both analyses.

### Detection of selection signatures

#### LSBL-based selection signatures in Arp populations

**Fig. 4:**
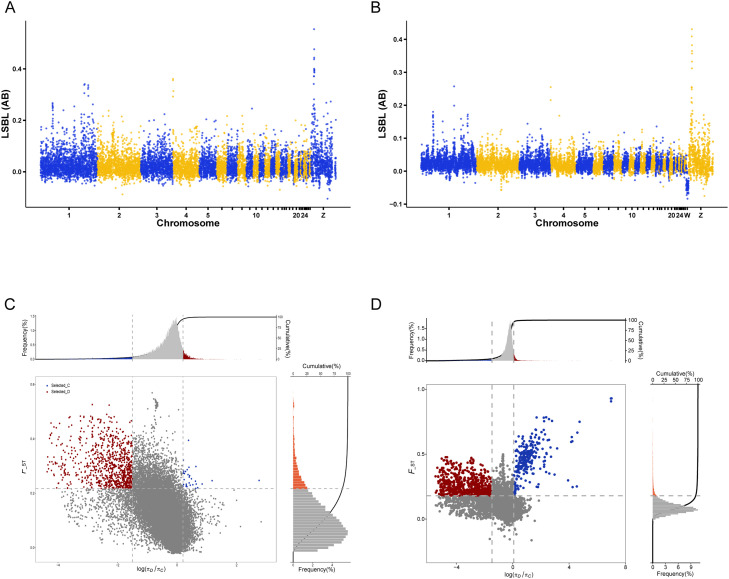
Selection signature analyses based on LSBL and joint *F*__ST_–π approaches. (A, B) Genome-wide LSBL distributions for Arp populations based on Shennong chip (A) and WGS (B). Alternating colors indicate different chromosomes. (C, D) Joint distribution of log₂(π ratio) and *F*__ST_ for RIR C vs. D based on Shennong chip (C) and WGS (D). Dashed lines indicate empirical thresholds, and colored points represent candidate regions under selection.

To identify genomic regions potentially subjected to selection in the Arp populations, window-based *F*__ST_ values were calculated for Arp vs RIR-C, Arp vs RIR-D, and RIR-C vs RIR-D comparisons, and locus-specific branch length (LSBL) values were then computed to emphasize branch-specific differentiation in the Arp population. Genome-wide LSBL Manhattan plots were generated (Fig. 4A–B), and windows within the top 5% of the empirical LSBL distribution were defined as candidate regions under selection (empirical threshold: LSBL ≥ 0.1077).

Comparable genome-wide patterns of LSBL signals were observed between Shennong chip–based and WGS-based analyses, supporting the robustness of the detected Arp-specific selection signals. The concordant LSBL signal profiles across platforms indicate that major Arp-specific differentiation signals can be consistently detected using Shennong chip dataset.

#### Joint analysis of nucleotide diversity and population differentiation between RIR C and D lines

To further characterize genomic differentiation within the RIR population, joint analyses of nucleotide diversity (π) and population differentiation (*F*__ST_) were performed between RIR lines C and D using both WGS and Shennong datasets (Fig. 4C-D). This approach aimed to identify genomic regions showing consistent divergence between the two parental lines.

Across both datasets, candidate regions were generally characterized by reduced nucleotide diversity (π) together with elevated *F*__ST_ values, indicating pronounced genetic differentiation between RIR C and D in these genomic windows. The overall genomic distribution patterns of π ratios and *F*__ST_ were largely consistent between the WGS- and Shennong chip–based analyses.

Compared with WGS, Shennong chip showed a narrower distribution of log-transformed π ratios and *F*__ST_ values, with fewer extreme signals detected. In particular, some divergence signals with relatively small genomic spans that were identifiable in the WGS analysis appeared attenuated in Shennong chip–based results. Nevertheless, major differentiation-related regions between RIR C and D remained consistently detectable across both platforms, indicating that Shennong chip was able to capture the primary genomic signals underlying population divergence within the RIR population.

#### Functional enrichment analysis based on Shennong chip–derived candidate regions

**Fig. 5:**
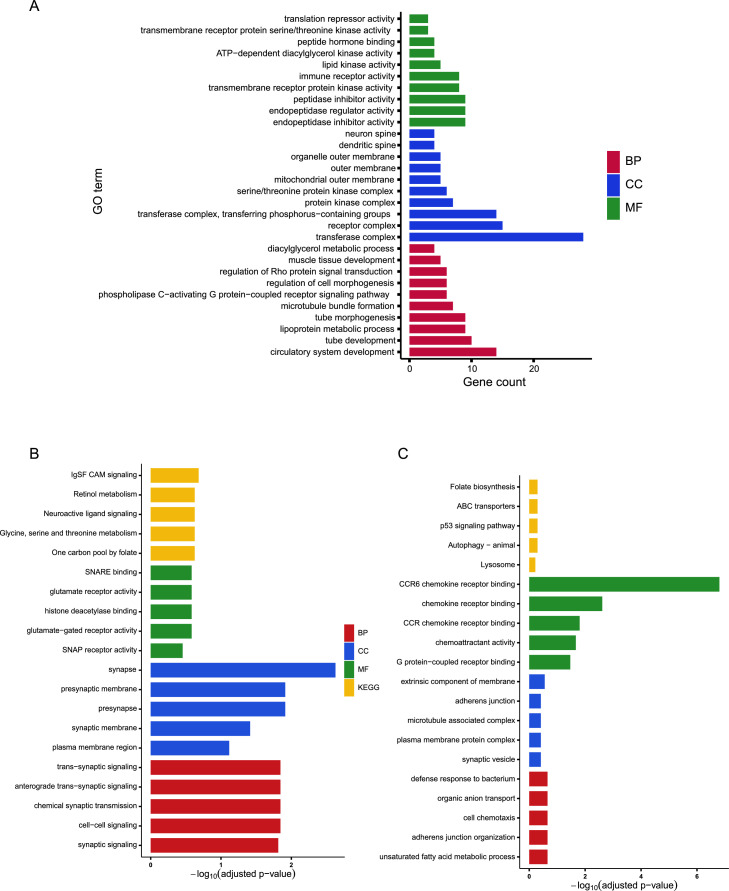
Functional enrichment of candidate genes identified from Shennong chip–derived selection signals. (A) GO enrichment of LSBL candidate genes in Arp populations. (B) Functional enrichment of candidate genes identified from RIR C–D divergence in line C. (C) Functional enrichment of candidate genes identified from RIR C–D divergence in line D. Bars are colored by category: BP, CC, MF, and KEGG. In panel A, the x-axis represents gene count, whereas in panels B and C, the x-axis represents −log₁₀(adjusted P value).

To evaluate whether Shennong chip–derived candidate regions capture biologically coherent signals, functional enrichment analyses were performed for genes annotated from (i) Arp candidate regions defined by LSBL(Fig. 5A), and (ii) divergent candidate regions between RIR lines C and D defined by *F*__ST_ and π ratio(Fig. 5B-C).

For Arp, GO enrichment analysis indicated (Fig. 5A) that candidate genes were mainly enriched for terms related to neural and synapse-associated functions, with enrichment signals largely concentrated in the Biological Process and Cellular Component categories. Under the applied significance thresholds, no significant KEGG pathway enrichment was detected for the Arp candidate gene set.

For the RIR C–D comparison, enrichment analyses revealed clear and contrasting functional signatures between the two parental lines (Fig. 5B-C). In RIR line C, the most significantly enriched GO terms were dominated by synapse- and neurotransmission-related categories, including synaptic signaling, chemical synaptic transmission, and synapse-associated cellular components (e.g., synaptic membrane and presynapse). Molecular function terms included glutamate receptor activity and SNARE binding, and KEGG pathways included Neuroactive ligand–receptor interaction among the top enriched categories.

In contrast, RIR line D showed enrichment primarily in metabolic, transport, and immune-related processes, including unsaturated fatty acid metabolic process, organic anion transport, cell chemotaxis, and defense response to bacterium. KEGG pathways highlighted Lysosome, Autophagy–animal, p53 signaling pathway, and ABC transporters among the top enriched categories.

## 3.Discussion

In this study, we conducted a systematic comparison between WGS and Shennong chip to evaluate the applicability of Shennong chip in commercial chicken populations. Using parental lines of Arp and RIR chickens as representative commercial breeding materials, we assessed population structure, genetic diversity, and selection signatures across the two genotyping platforms. Overall, Shennong chip yielded results that were highly consistent with those obtained from WGS for major genomic patterns, indicating that it represents a reliable and practical tool for genomic analyses in commercial chicken breeding populations.

Population structure analyses based on PCA, phylogenetic reconstruction, and ancestry inference consistently revealed clear genetic differentiation between RIR C and RIR D, whereas Arp A and Arp B showed substantial overlap and strong genetic homogeneity. Importantly, these patterns were reproducible across both Shennong chip and WGS datasets, suggesting that they reflect genuine biological structure rather than platform-specific artifacts (Gao et al., 2023). A small number of individuals from the RIR D line deviated from the main cluster in both PCA and phylogenetic analyses. Because this pattern was consistently observed across both WGS- and Shennong chip–based analyses, it is unlikely to result from platform-specific technical noise. Instead, it may reflect underlying genetic heterogeneity within the D line, potentially arising from historical introgression, cryptic admixture, or differences in selection and population management during line development (Li et al., 2019). Importantly, these outlier individuals represented only a small proportion of the samples, and the overall patterns of population structure and genetic diversity remained stable, suggesting that their presence did not substantially affect the main conclusions of this study. As no additional validation was performed in the present study, the precise origin of these outliers remains to be clarified in future work integrating pedigree information and expanded genomic data (Xu et al., 2023). As with other medium-density SNP arrays, the Shennong chip may be affected by ascertainment bias because marker selection generally favors common and informative variants. Consequently, rare and population-specific variants may be underrepresented, which could lead to slight underestimation of genetic diversity and reduced sensitivity for detecting fine-scale or weak selection signals compared with WGS. Nevertheless, in the present study, the Shennong chip remained highly consistent with WGS in major population structure patterns and broad selection signatures.

ADMIXTURE analyses further supported the robustness of Shennong chip–based population inference. At lower K values (K = 2–3), ancestry patterns inferred from Shennong chip were highly consistent with those derived from WGS, clearly separating Arp from RIR and reliably distinguishing RIR C from RIR D (Decker et al., 2009). Differences between the two platforms became apparent only at higher K values (K ≥ 4), where WGS revealed additional fine-scale substructure, whereas Shennong chip produced smoother and more consolidated ancestry profiles. These differences are best interpreted as a consequence of intrinsic differences between the two genotyping strategies rather than discrepancies in analytical accuracy. WGS captures a dense spectrum of genetic variants, including rare and population-specific SNPs, which increases sensitivity to subtle within-line structure (Patterson et al., 2006). In contrast, SNP chip markers are typically selected for higher minor allele frequencies and broad informativeness across populations, allowing stable resolution of major population structure while reducing noise introduced by low-frequency variants. From an applied perspective, this characteristic makes the SNP chip particularly suitable for routine population monitoring and long-term consistency assessment in commercial breeding programs (Kranis et al., 2013), whereas WGS remains advantageous for fine-scale demographic and evolutionary analyses.

From an applied perspective, these differences highlight the complementary nature of Shennong chip–based genotyping and WGS (Albrechtsen et al., 2010; Nielsen et al., 2011). The relatively smooth ancestry patterns produced by the SNP chip at higher K values suggest that this platform is particularly well suited for routine applications such as breed identification (Alexander et al., 2009), assessment of population structure stability, and long-term genetic monitoring in commercial breeding populations (Malomane et al., 2018; Matukumalli et al., 2009). By contrast, WGS offers greater resolution for detecting subtle population substructure and is therefore more appropriate for fine-scale analyses, reconstruction of evolutionary history, and investigation of complex admixed populations. In this context, the two technologies should be viewed as complementary tools rather than direct alternatives (Wiggans et al., 2017).

Selection signature analyses further demonstrated that biologically meaningful signals can be detected using Shennong chip data. LSBL-based analyses identified candidate regions under selection in Arp populations, and comparable genome-wide signal distributions were observed between Shennong chip- and WGS-based analyses. Functional annotation of Arp LSBL candidate regions revealed enrichment primarily in neural- and synapse-related Gene Ontology categories, particularly within Biological Process and Cellular Component terms. Representative genes contributing to these enrichments included synapse-associated and signaling-related genes such as *GRIA2* (Isaac et al., 2007) and scaffold-related genes such as *DLGAP1* (Welch et al., 2007). Under the applied significance thresholds, no significant KEGG pathway enrichment was detected for Arp, suggesting that selection in this population may act through distributed regulatory mechanisms rather than strong pathway-level aggregation.

In contrast, joint analyses of population differentiation (*F*__ST_) and nucleotide diversity (π) between RIR C and RIR D revealed clearer and more functionally interpretable divergence patterns, which were consistently captured by both Shennong chip and WGS datasets. Functional enrichment analyses based on Shennong chip–derived candidate regions highlighted distinct selection orientations between the two parental lines. In RIR C, enriched genes were predominantly associated with neural signaling and synaptic regulation, including synaptic transmission, neurotransmitter receptor activity, and synapse-associated cellular components. Representative genes such as *GRIK1* (Cui et al., 2012), *GRIN3A* (Pérez-Otaño et al., 2016), *SNAP47* (Arendt et al., 2015), and *CDH2* (Ferreira et al., 2023) are involved in glutamatergic signaling, synaptic vesicle trafficking, and cell–cell adhesion, respectively(Zhang et al., 2026). These functions are closely linked to behavioral regulation, feeding behavior, and central nervous system integration of environmental cues, suggesting that selection in the C line may be more strongly associated with growth- and behavior-related regulation, although direct behavioral phenotypes were not assessed in the present study.

By comparison, RIR D showed enrichment primarily in metabolic, transport, and immune-related processes, including lipid metabolism, organic anion transport, lysosomal function, autophagy, and defense response to bacterium. Representative genes included multiple members of the SLC transporter family (Obinata et al., 2025; Sadr et al., 2025), *ABCA*/*ABCB* transporters (Alam and Locher 2023), and avian β-defensin (*AvBD*) genes (Lyu et al., 2020). KEGG pathway analysis highlighted Lysosome, Autophagy – animal, ABC transporters, and the p53 signaling pathway among the most significantly enriched categories. These functional themes are closely related to nutrient utilization efficiency, metabolic homeostasis, and immune robustness, suggesting that the D line may have experienced stronger selection pressure related to feed efficiency and resilience under intensive production conditions (Xiao et al., 2021).

Taken together, the contrasting functional enrichment patterns observed between RIR C and RIR D are consistent with their roles as complementary parental lines in commercial breeding systems, where different selection emphases are applied to optimize overall crossbred performance. Although Shennong chip detected fewer extreme signals than WGS—likely due to its marker composition and reduced sensitivity to rare variants—it consistently captured the major genomic regions and core functional themes underlying population divergence and selection. Overall, our results demonstrate that the Shennong chip can be reliably applied to commercial chicken populations, providing population structure inference, selection signal detection, and biologically interpretable functional insights that are highly consistent with WGS, while offering clear advantages in cost efficiency, analytical stability, and scalability for routine breeding applications.

## Conclusions

Shennong chip shows high consistency with WGS in resolving population structure, genetic diversity, and major selection signals in commercial chicken populations. Using Arp and RIR parental lines as examples, the Shennong chip can reliably distinguish homogeneous and divergent breeding lines and capture key genomic differentiations. Analysis of selection signals between the RIR C and RIR D lines reveals distinct breeding goals: the RIR C line is enriched for neural signaling-related genes (e.g., *GRIK1*), whereas the RIR D line shows enrichment for metabolic and immune-related genes (e.g., *AvBD* family members).

These results indicate that the Shennong chip provides a cost-effective, stable, and scalable alternative to WGS for routine population monitoring, line differentiation, and selection evaluation in commercial chicken parent lines such as Australorp and Rhode Island Red. Its applicability to other commercial chicken breeds and hybrid populations still requires further validation.

## Declaration of competing interest

The authors declare no conflict of interest.

## Data avail ability statement

The data generated in this study are publicly available and can be obtained from the corresponding author upon reasonable request.

## CRediT authorship contribution statement

**Zhao Cai:** Writing – original draft, Visualization, Formal analysis. **Shu Wang:** Data curation. **Chaoqun Gao:** Formal analysis. **Chenpeng Xiao:** Formal analysis. **Yulong Ma:** Formal analysis. **Yunjuan Liu:** Data curation. **Kejun Wang:** Visualization. **Yadong Tian:** Visualization. **Xuewei Fan:** Data curation. **Hangran Gao:** Data curation. **Guirong Sun:** Visualization. **Xiangtao Kang:** Conceptualization. **Wenting Li:** Writing – review & editing, Conceptualization.

## Disclosures

The authors declare that they have no competing financial interests with the study.
